# Ergonomic interventions to reduce upper limb musculoskeletal pain during robotic surgery: a narrative review

**DOI:** 10.1007/s11701-024-01992-w

**Published:** 2024-05-27

**Authors:** Shing Wai Wong, Allan Parkes, Philip Crowe

**Affiliations:** 1 Randwick Campus, School of Clinical Medicine, The University of New South Wales, Sydney, NSW Australia; 2 Department of General Surgery, Prince of Wales Hospital, Sydney, NSW Australia

**Keywords:** Ergonomics, Musculoskeletal pain, Robotic surgery, Upper limb

## Abstract

There is a high prevalence of upper limb musculoskeletal pain among robotic surgeons. Poor upper limb ergonomic positioning during robotic surgery occurs when the shoulders are abducted, and the elbows are lifted off the console armrest. The validated rapid upper limb assessment can quantify ergonomic efficacy. Surface electromyography and hand dynamometer assessment of strength are the most common methods to assess muscle fatigue. A literature review was performed to find evidence of ergonomic interventions which reduce upper limb musculoskeletal pain during robotic surgery. There is a paucity of studies which have reported on this topic. In other occupations, there is strong evidence for the use of resistance training to prevent upper extremity pain. Use of forearm compression sleeves, stretching, and massage may help reduce forearm fatigue. Microbreaks with targeted stretching, active ergonomic training, improved use of armrest, and optimal hand controller design have been shown to reduce upper limb musculoskeletal pain. Future studies should assess which interventions are beneficial in reducing surgeon upper limb pain during robotic surgery.

## Introduction

Ergonomics is the study of workplace design with the aim of optimising performance and minimising injury. There is a high prevalence of musculoskeletal pain among surgeons. Pain induced by poor ergonomics was found to be widespread across multiple surgical subspecialties and has been attributed to poor knowledge and access to ergonomic furniture [1]. Reducing work-related injuries through ergonomic interventions is important to ensure longevity of surgeons’ careers and to maximise comfort and efficiency.

Ergonomic principles postulated by Fernandez include preparation for dynamic rather than static work, working within 30% of maximum voluntary contraction, placement of primary controls within normal working area, use of both hands, minimisation of finger overload, and avoidance of unnatural posture with regular adjustments [2]. In the operating room, awkward postures, static positions, repetitive muscle use, and forceful exertions can increase the risk of injury [3, 4].

Although accuracy of injury statistics among surgeons are hindered using survey methodology which are prone to volunteer or recall bias, the impact of musculoskeletal injuries is likely to be significant. Davis et al. found that 54% of surgical injuries had an impact on work with 22% resulting in missed work of an average duration of 7 days per injury [5]. Even outside of missed work, musculoskeletal injuries greatly impact surgeons by reducing their quality of life. 80% of surgeons self-reported having experienced pain in the last 24 h and 47% of surgeons were concerned that musculoskeletal pain may shorten their career [6, 7].

A review by Catanzarite et al. found concerning rates of injuries for all surgical disciplines with 66–94% for open surgery, 73–100% for conventional laparoscopy, and 23–80% for robotic-assisted surgery [8]. Minimally invasive surgery may be less physically demanding overall compared with open surgery but may result in more static posture and low-level muscle tension [9]. Robotic surgery (RS) may offer improved ergonomics over laparoscopic surgery (LS). Studies assessing fatigue in the different upper limb muscle groups of surgeons during RS and LS have reported conflicting results. The greatest muscle strain has been reported to occur in the forearms during RS and in the shoulders during LS [10–12]. A meta-analysis revealed the biceps as the only muscle group that consistently demonstrated significantly lower muscle activation for RS when compared with LS [13]. The ability to rest the forearms on the robotic console bar may explain the results of less shoulder discomfort and less biceps activation. The other potential benefit of robotic surgery in relation to upper limb ergonomics involves ambidexterity. It has been shown that the muscle activity in forearm and shoulder muscles was significantly greater on the dominant (right) side compared with the non-dominant (left) during LS but not during RS [11].

One non-simulated study found a reduction in dexterity and more hand muscle fatigue after two hours of LS, as measured by the Purdue Pegboard Test and the Camry Electronic Handgrip Dynamometer, in contrast to no difference after 2 h of RS [14]. Robotic upper gastrointestinal surgery resulted in less musculoskeletal stress to the upper extremities than standard laparoscopic technique as measured with rapid upper limb assessment (RULA) score in a single-surgeon study [15]. However, a randomised control trial of 102 surgeons found no difference in upper limb ergonomics as measured by the RULA instrument when performing robotic versus laparoscopic inguinal hernia repair [16].

Poor upper limb ergonomic positioning during RS occurs when the shoulders are abducted, and the surgeons’ elbows are lifted off the console armrest [17]. This can be avoided by use of the clutch control system to reposition the control manipulators back to a neutral position [18]. Frequent judicious clutching can return the forearms to the neutral position with the elbows at 90 ℃, tucked in, adjacent to the torso, and resting comfortably on the armrest [19–21]. Sustained forward reach posture during manoeuvring of the controls was observed to be an ergonomic risky behaviour during RS [1]. Non-ergonomic positioning of the hands at the robotic console away from the central position with the shoulders abducted occurred more frequently with instrument tip manipulation in the peripheral rather than central working envelope on the robotic screen [22].

Use of forearm compression sleeves, rest breaks, and prevention through conditioning may help reduce forearm fatigue [19, 20, 23]. Microbreaks with targeted stretching during surgery can interrupt long-lasting periods of low-level intensity and has been shown to significantly reduce post-procedure musculoskeletal pain for laparoscopic and open procedures [7]. RS has the advantage of allowing the ungowned surgeons more freedom to stretch as long as they are mindful to take a break from their immersive robotic console environment.

Most studies on ergonomic interventions to reduce upper limb musculoskeletal pain have been performed on office workers, athletes, and surgeons performing open or laparoscopic surgery. There is a paucity of research investigating ergonomic interventions in RS partly because it has been a relatively new technique. This narrative review will present the evidence of optimal upper limb positioning, assessment of upper limb ergonomics, and ergonomic interventions which may be applicable to robotic surgeons. We will extrapolate findings from other subject groups if research of certain ergonomic interventions during RS are lacking and suggest recommendations.

## Methods

A literature search was conducted by two authors (SW and AP) independently in February 2024, using MEDLINE (Pubmed). Studies focussing on ergonomic interventions to prevent or reduce upper limb musculoskeletal pain during robotic surgery, either real or simulated, were reviewed. Studies focussing on back and lower limb pain and animal studies were excluded. All studies published from inception to February 2024 were assessed and no restrictions were imposed regarding study design. Original English language articles published in peer-reviewed journals were considered. To find relevant publications, keywords ‘ergonomics’, ‘robotic surgery’, ‘upper limb’, and “pain” were used. Other keywords were extracted from interventions used to reduce upper limb musculoskeletal pain in robotic or laparoscopic surgery. These keywords were “forearm sleeves”, “strengthening”, “stretching”, “massage”, “microbreaks”, “ergonomic training”, “armrest” supports”, and “hand controller”. In addition, a search of relevant references cited in the studies and reviews was conducted to include literature which were not discovered on initial search. Initially, research on optimal ergonomic upper limb positioning and their assessment are presented. Subsequently, the narrative review presents the different interventions as subheadings with discussion of the relevant information from the studies.

## Discussion

### Optimal upper limb positioning

One study, which included consultation with an upper body biomechanics ergonomic expert as well as a literature review of ergonomic guidelines for use of the daVinci^©^ robotic system, recommended seated positioning with the knees at a 90° angle or greater, upper arms perpendicular to the floor with the elbows forming a 90° angle and tucked close to the body, and the forearms resting on the armrest [17]. Placement of the forearms on the console bar has been recommended by many authors, and can reduce fatigue and improve the precision of forearm movements [21, 22]. Supporting the weight of the upper limbs reduces the validated RULA posture score [24].

Experienced robotic surgeons can achieve a significantly lower workspace range by separating long movements into short ones with frequent use of the clutch. Clutching of the control manipulators maintained the elbows in a neutral position, avoided shoulder abduction, and reduced tension on the intrinsic hand muscles [20]. Studies have shown clutch control use to be more frequent with initial coaching and increasing robotic experience [25–27]. Coaching instructions on clutch usage discouraged removing the forearms from the console armrest to prevent the start of a cascade of movements, which can lead to overexertion of the arms and rotation of the trunk [26].

### Assessment of upper limb ergonomics

Ergonomics and biomechanics may be objectively measured using the validated rapid upper limb assessment (RULA) [24]. The RULA posture scores are related to the angulation of the shoulder, elbow, and wrist in the sagittal plane; with extreme postures associated with extra load. In addition, shoulder abduction or elevation, lower arm working across the midline of the body or out to the side, and radial or ulnar deviation of the wrist all add an extra point to the posture score. Supporting the weight of the arm results in subtraction of a point to the posture score. The RULA score incorporates the posture score, muscle use (additional load score with excessive static muscle work or repetitive motions), and force score (requirement to maintain an external load while working). The average RULA score for four surgeons during robotic surgery was 4.75 (out of a maximum score of 7) in one study, where a score of 1–2 represented acceptable posture, 3–4 need for further investigation and change, 5–6 need for prompt investigation and change, and 7 immediate investigation and implementation of change [28]. Another study reported a mean RULA score of 6.5 during robotic gynaecology surgery and also recommended a need for modification and intervention [29].

The armrest load has been used for ergonomic assessment because it has been demonstrated to be significantly higher in experts compared with novices during simulated robotic exercises [21]. The pressure surveillance system in this study used force-sensing resistors to measure three metrics: maximum time the arms were not on the armrest, the total number of times arms left the armrest, and the touching rate (time of touching the armrest divided by the total time of exercise) [21].

Surface electromyography (EMG) is the preferred and most objective method for quantitative assessment in studies assessing fatigue in different muscle groups [30]. Motor fatigue can also be measured with a quantitative grip dynamometer. Handgrip strength assessment (with repeat measurements via a hand dynamometer) has been shown to be a sensitive tool to assess muscle fatigue [9, 31].

### Forearm compression sleeves

There have been no studies investigating the potential benefit of forearm compression sleeves during RS, especially when there is no prohibitive reason for its usage related to the maintenance of sterility. Compression garments are a popular intervention amongst both recreational and elite athletes to improve performance, reduce risk of injury or mitigate discomfort. Forearm compression sleeves may reduce fatigue and improve muscle recovery via better muscle oxygenation [32–34]. A meta-analysis suggested that compression garments may aid in the recovery of exercise-induced muscle damage [35]. There was good evidence that compression garments reduced perceived muscle soreness and swelling, without decreasing serum lactate or creatine kinase levels. A review of the evidence found that while compression garments appear to have positive impacts, the research papers demonstrated great heterogeneity in both methods and results—with variability including nature of the exercise, measures of exercise and recovery performance, and timing and duration of garment wear [36]. A review of 23 randomised controlled trials found no relationship between the amount of pressure applied and effectiveness of recovery after exercise [37].

### Strengthening, stretching, and massage

A few studies on ergonomics and robotic surgery offered exercise or posture recommendations but did not analyse the effects of any active intervention [18, 23, 38]. There is limited evidence of effectiveness of exercises for surgeons. General exercise has been shown to have a dose-related protective effect against pain in a web-based survey of 701 urology surgeons [39]. In a multicentre randomised controlled trial of surgeons, Giagio et al. found that self-treatment physical exercises supervised by a physical therapist (which included non-resistance active exercises and static stretching) resulted in a statistically significant improvement of symptoms in the lower back at 6 months (non-training: 72% vs training: 53%; *P* = 0.04) [40]. However, statistical significance was not reached for the upper limb areas of shoulder, elbows, and wrists/hands (*P* = 0.08, 1.00, and 0.52).

Resistance strengthening exercises, muscle stretching, proprioception training, and massage have been shown to reduce fatigue and pain in the upper extremities of workers [41]. With other occupations, a systematic review of workplace interventions to prevent upper extremity pain found strong evidence for the use of resistance training and no benefit with office workstation adjustment [42]. Seven studies found a positive effect from resistance exercises using dumbbells or kettlebells. A narrative review found that resistance-training programs through adulthood reduced musculoskeletal disorders by attenuating loss of muscle strength [43]. For sports injuries, consistently favourable outcomes were obtained for all injury prevention measures except with stretching exercises. In a meta-analysis of 25 randomised controlled trials, strength training demonstrated a trend towards better preventive effect than proprioception training, compared with no training (relative risk 0.351 and 0.480) [44]. Delayed-onset muscle soreness may be preventable with massage or active exercises [45–47]. Reduction of pain associated with massage may be related to decreased creatine kinase enzyme or psychological reasons [48].

### Microbreaks with targeted stretching

Studies have shown that uninterrupted periods of low-level activity lasting more than 4 min to be associated with the development of musculoskeletal pain [49]. Microbreaks with targeted stretching during surgery can interrupt long-lasting periods of low-level intensity and has been shown to significantly reduce post-procedure shoulder musculoskeletal pain for laparoscopic and open procedures [6, 7]. Robotic surgery may allow surgeons more freedom to stretch because of fewer restrictions on maintaining sterility. A systematic review of four studies investigating the use of microbreaks during the surgery found significantly improved self-reported pain in the shoulders and error rates, but conflicting results with regards to improved accuracy [50].

In a four-centre cohort study involving 61 surgeons, Park et al. investigated the effects of ‘micro-breaks’ (1.5–2 min) with targeted stretching during surgeries at appropriate 20–40 min intervals [7]. Intervention was associated with improved pain scores, perceived physical performance, and improved mental focus. 87% of the cohort stated they planned to continue using the microbreaks. In a four-centre cohort study of 56 surgeons by Hallbeck et al., 88% of surgeons reported improvement or no change in their mental focus and significantly reduced shoulder discomfort with introduction of microbreaks [6]. In a prospective crossover study of 16 surgeons performing at least 2 h non-simulated surgery, Dorion and Darveau found that 20 s microbreaks every 20 min had statistical and clinical significance in reducing fatigue from surgery [51]. Furthermore, Engelmann et al. tested stress hormones and α-amylase in surgeons’ saliva and found that regular intraoperative breaks during laparoscopic surgery reduced markers of stress in surgeons, potentially optimising performance [52].

There was moderately strong evidence for the use of microbreaks to prevent upper limb pain in a systematic review of health professionals which included surgeons [53]. A Cochrane review investigating prevention of upper limb pain in office workers found benefit with the implementation of breaks 2 studies [54].

### Ergonomic training and positioning

Maintenance of a neutral posture has been advocated as an important ergonomic guideline for surgeons [3]. Improving the posture of the robotic surgeon by a trained ergonomists was shown to improve objective right upper arm posture as measured by inertial measurement unit sensors during 18 real hysterectomy surgery performed by six surgeons when compared with the surgeons’ self-selected settings [55]. Having an ergonomic expert adjust robotic console settings from surgeon self-selected settings resulted in a significant decrease in the time spent in moderate- to high-risk areas for the right upper arm (mean, 15.5% vs 0.9%; *p* = 0.02) [55]. The ergonomic intervention protocol involved adjusting the chair height to match the popliteal height, moving the chair as close to the console armrest as close as possible, adjusting the console height to the highest level where the surgeon could use the binoculars comfortably, moving the armrest height 2–3 inches above the level when the shoulders were relaxed, and finally moving the pedal location to a position where the surgeon’s knees were at right angles.

In a two-part survey study of 42 residents, fellows, and surgeons, Franasiak et al. found that robotic surgeons noted a reduction in self-reported pain after implementation of either an online or in-person ergonomic training session which included adjustment of the armrest to allow forearm positioning parallel to the floor (which was changed in 68% of case) and intermittent stretching [20]. Frequent clutching of the control manipulators to maintain the elbows in a neutral position was recommended. This position avoided excessive and sustained shoulder abduction and internal rotation, and reduced tension of the intrinsic hand muscles. Another study of 26 junior doctors also found that ergonomic instructions prior to simulation robotic surgery improved postures as measured by RULA score but did not significantly improve physical comfort [26]. Optimal armrest height was found to be outside the adjustment range of the console for the da Vinci^©^ robot in short and very tall surgeons [26].

Dalager et al. found no consistent or clinically significant difference in trapezius muscular activity in six surgeon subjects as measured by EMG or total RULA scores with use of an ergonomic chair [56]. The first ergonomic chair had a backrest, and the second ergonomic chair had a smaller castor and smaller seating.

Ergonomic training programs are uncommon. Wauben et al. reported that 89% of surgical respondents were unaware of ergonomic guidelines [57]. Park et al. found that 59% of surgeons reported being slightly or not aware of ergonomic recommendations [58]. Although heterogeneous in results, these studies indicate a picture of proper ergonomic training being uncommon among surgeons. However, ergonomic knowledge and training has been demonstrated to be highly effective in reducing symptoms of workplace injuries amongst surgeons [40, 50].

The aim of active ergonomic training is to improve the workers’ awareness of proper ergonomic practices. It has been shown to reduce upper limb pain in a 12-month randomised controlled study involving administrative workers [59]. The active ergonomic training included attendance at a full-day course, individual workplace visit 1 month later which included a counselling session and adjustment of the workstation, and provision of a working posture checklist to be adhered to.

The Alexander technique (AT), which involves body position mindfulness, rediscovering natural balance and poise through thinking in activity, and gentle movements using only the appropriate amount of muscular energy, was used in one study of seven subjects performing laparoscopic surgery [60]. The AT training program resulted in subjective improvement in posture, decreased discomfort, and decreased intentional tremor score of the non-dominant hand [60]. The proposed benefit may relate to improving core stability to achieve better co-ordination and balance.

### Armrest supports and pressure sensors

An upper limb ergonomic advantage of the robotic console is the presence of the armrest, which can reduce weightbearing and static load [18]. The armrest load has been shown to be significantly higher in 5 experts compared with 48 novices during simulated robotic exercises [21]. The authors found that novices tended to raise their arms when they needed to reach a target on the periphery of the operative field or when they changed the instrument’s direction. There is potential for the robotic surgeon to receive continuous feedback during surgery from pressure sensors on the armrest to help reduce upper limb musculoskeletal pain [21]. A laparoscopic surgery study revealed improvement of ergonomics with the use of an arm support system [61]. The authors found that supporting the elbow from below resulted in lower trapezius activity and was favoured over two other upper limb support systems (cable support for the forearm and pneumatic vest support for the upper arm) by the surgeons.

Previous reviews have reported evidence of benefit with the use of forearm supports in non-surgery associated work [42, 62]. There was moderately strong evidence for the use of armrest supports to prevent upper limb pain in a systematic review of health professionals which included surgeons [53]. A Cochrane review investigating prevention of upper limb pain in office workers found inconsistent results with the use of arm supports 3 studies [54].

### Hand controller design

With the da Vinci^©^ robot, the thumbs and index fingers are placed in the loops of the hand controllers and moved in a pinching motion intuitively to mimic the end-effector movement [63]. In a survey of 1215 surgeons who performed open, laparoscopic, and robotic surgery, robotic surgery was reported to be more likely to cause thumb and finger pain than open surgery [64]. Prolonged pinching of the thumb and index finger can lead to temporary neuropraxia/numbness [64, 66]. The surgeon can opt to place their fingers outside rather than inside the loops which will allow a wider range of wrist pronation/supination movement but a potential higher risk of slippage. The larger contact area can help distribute the pulp pressures more widely.

A previous study reported on eight ergonomic considerations for the design of laparoscopic instrument handles [66]. The ergonomic requirements which are relevant (and possibly modifiable) with the robotic system include the ring dimensions, presence of spring, big contact area, and little opening/closing force required. Other robotic systems use hand controls that resemble traditional laparoscopic instrument controls (resulting in ergonomic problems similar to those experienced with laparoscopic instrument handles use) and a hand-grip system [63]. Other alternative handle designs for robotic neurosurgery have been proposed [67, 68]. Use of a robotic handle mounted to the forearm which mapped the surgeon’s wrist movement directly to the robot joints resulted in improved dexterity, acceptable RULA scores, and less fatigue [67]. An alternative robotic instrument handle design for endoscopic endonasal skull base surgery, utilising a rotating joystick-body handle, was reported to be associated with an improvement in both performance and ergonomics compared to the standard tool [68].

## Conclusion

There have not been many published studies on ergonomic interventions which may reduce upper limb musculoskeletal pain during robotic surgery. Most studies on this topic have been performed during simulation rather than real surgery. The use of armrests and more freedom to take breaks during robotic surgery are two factors which should help reduce surgeon upper limb pain. Use of compression arm sleeve, strengthening exercises, massage, and conscious reminders to use the clutch to maintain the upper limbs in neutral position are simple interventions to implement and have the potential to reduce occupational injury and prevent disability. Table 1 summarises the effectiveness of the ergonomic interventions. Future studies can assess whether these interventions are beneficial in robotic surgery.

**Table 1:** Ergonomic interventions to reduce upper limb musculoskeletal pain

Intervention	Study	Type of study	Participant characteristic	Study or subject numbers	Results	Effect
Forearm sleeve	Marques-Jimenez et al. MacRae et al. Beliard at al.	Meta-analysis (20 studies) Review Review of RCTs	Variety Variety Variety	9 studies 5 studies 2 studies 5 studies 19 studies 23 studies	Creatine kinase no change Lactate increased Lactate dehydrogenase decreased Muscle swelling decreased Studies too heterogenous No relationship between pressure and effect	+ + ~
Strength training	Lloyd et al. Giagio et al. Van Erd et al. Ciolac et al. Lauersen et al.	Web-based survey MC RCT Systematic review Narrative review Meta-analysis	Urologists Surgeons Variety Variety Sportspeople	701 141 26 studies 25 studies	Dose-related protective effect with general exercises No significant improvement for upper limbs Strong evidence for resistance training Reduced MSK disorders Strength training and proprioceptive training effective	+ ~ ++ + ++
Microbreaks	Park et al. Hallbeck et al. Dorion et al. Engelmann et al. Sweeney et al. Hoe et al.	MC RCT MC RCT Prosp crossover RCT Systematic review Cochrane review RCTs	Surgeons Surgeons Surgeons Laparoscopic surgeons Sonographers, surgeons, dentists Office workers	61 56 16 7 (51 cases) 31 studies 15 studies	Improved pain scores and mental focus Significantly reduced shoulder pain Reduced fatigue Significantly fewer intraoperative events Moderately strong evidence of reduced UL pain May reduce UL discomfort (2 studies)	++ ++ ++ ++ ++ +
Ergonomic training	Hokenstad et al. Franasiak et al. Van’t Hullenaar et al. Dalagher et al. Reddy et al.	Prosp Robotic Prosp Robotic Prosp Robotic Prosp Robotic Prosp cohort	Gynaecology surgeons Residents, fellows, surgeons Junior doctors Surgeons Trainee surgeons	6 42 26 6 7	Improved objective UL posture Reduction in self-reported pain Improve RULA score nut not physical comfort No difference in shoulder muscular activity with ergonomic chair Alexander technique improved posture and decreased discomfort	+ + ~ − +
Armrest support	Yang et al. Steinhilber et al. Sweeney et al. Hoe et al.	Prosp Robotic Prosp Systematic review Cochrane review RCTs	Surgeons and novices Laparoscopic surgeons Sonographers, surgeons, dentists Office workers	4 and 48 3 31 studies 15 studies	Simulation study—armrest load significantly higher in experts Less shoulder muscle activation with arm support Moderately strong evidence to reduce UL pain Inconsistent results	++ + + ~
Hand controllers	Plerhoples et al. Dimitrakakis et al. X2	Survey Prototype RCT	Surgeons Surgeon Novice	1215 1 9	More thumb and finger pain with robotic surgery Alternative robotic handle improves performance	− +

*MC* multicentre, *RCT* randomised controlled trials, + positive, ~ no change, − negative, *MSK*  musculoskeletal, *Prosp* prospective, *UL* upper limb, *RULA* rapid upper limb assessment

## Data Availability

No datasets were generated or analysed during the current study.
